# Knowledge of obstetric danger signs and birth preparedness and complications readiness among mobile Pokot nomadic pastoralist pregnant women in Tiaty Sub-County, Baringo County-Kenya

**DOI:** 10.1186/s12884-025-07532-0

**Published:** 2025-05-10

**Authors:** Evans Kasmai Kiptulon, Dahabo Adi Galgalo, Mohammed Elmadani, Mate Orsolya, Adrienn Ujváriné Siket

**Affiliations:** 1 Doctoral School of Health Sciences, Faculty of Health Sciences, University of Pécs, Pécs, Hungary; 2 Doctoral School of Health Sciences, Faculty of Health Sciences, University of Debrecen, Debrecen, Hungary

**Keywords:** Baringo, Birth preparedness and complication readiness, Mobile nomadic Pokot pastoralists, Obstetric danger signs, Pregnant women, Tiaty sub-county

## Abstract

**Background:**

Maternal mortality in Kenya remains unacceptably high. Mobile pastoralist communities, Pokot community included take the greatest burden of these maternal deaths. Knowledge of obstetric danger signs is important for the prevention of pregnancy and birth complications as it influences pregnant women to prepare for birth and complications. The aim of this study was to assess the level of knowledge of obstetrics danger signs and their effect on Birth Preparedness and complications Readiness among mobile Pokot nomadic pastoralist in Kenya.

**Methods:**

A descriptive cross-sectional study was conducted among 275 pregnant Pokot nomadic pastoralist women using a multistage sampling technique. Chi-square (X^2^) was used to test the association between categorical variables and a *P*-value of < 0.05 was considered significant.

**Results:**

Majority of the women demonstrated a high level of knowledge regarding obstetric danger signs, with 80% (*n* = 220) recognizing these danger signs during pregnancy and 69.1% (*n* = 190) during labour. However, despite this awareness, the overall Birth Preparedness and Complication Readiness (BPCR) remained low, with only 28% (*n* = 77) adequately prepared and 77% (*n* = 212) were unprepared.

**Conclusion:**

High awareness of obstetric danger signs among Pokot pastoralist women has not translated into adequate birth preparedness and complication readiness. To reduce maternal mortality especially in these pastoralists and unsettled communities where services are unpredictable and disrupted by frequent movements, government and other stakeholders must implement powerful, targeted actions that address their unique challenges such as robust mobile clinics and outreach services, community based birth preparedness programs, emergency transport networks, maternity waiting homes, improved referral systems, culturally sensitive health education and policy and infrastructural investments. These bold, community-centered interventions can significantly reduce maternal deaths and improve maternal health outcomes among Kenya’s pastoral populations.

## Background

Obstetric Danger Signs (ODS) are warning signs that women encounter during pregnancy, childbirth, and post-partum periods that indicate imminent or ongoing life-threatening events requiring quick intervention by skilled healthcare providers [1]. Danger signs are not actual obstetric complications, but symptoms that can easily be identified by non-clinical personnel [2–4]. During pregnancy, these danger signs are vaginal bleeding, severe headache, blurred vision, convulsions, swollen hands, legs, or face, high fever, loss of consciousness, difficulty breathing, severe body weakness, severe abdominal pain (false labour), and reduced fetal movement. During delivery, they include excessive bleeding, severe headache, convulsions, fever, loss of consciousness, long hours of labour, and retained placenta ([5–7].

Maternal mortality (MM) in Kenya has continued to be a major public health problem [8–10]. Maternal mortality in the country remains unacceptably higher compared to other Sub-Saharan African countries like South Africa, Angola, and Botswana which have less than 200 maternal deaths per 100,000 live births. The reduction of maternal mortality ratio (MMR) has been one of the slowest processes [9]. According to the Kenya Demographic Health Survey 2014, Kenya's MMR was 362 deaths per 100,000 live births compared to 488 per 100,000 deaths in 2008 [11, 12]. There are a lot of discrepancies in these maternal deaths per county and region in Kenya with most counties occupied by pastoralists taking the greatest burden [13]. Mandera County, for example, has the highest deaths of over 1,000 deaths per 100,000 live births [14] and the lowest 189/100,000 in Elgeyo Marakwet County [15]. The top five counties with high MMR are counties occupied by pastoralist communities. These include Mandera, Wajir, Turkana, Marsabit and Isiolo. Only 15 out of 47 counties account for 98.7% MMR with West Pokot and East Pokot regions among these 15 regions [16].

Maternal morbidly and mortality could be prevented significantly if women and their families recognize obstetric danger signs and promptly seek health care [3, 7, 17–19]. Knowledge of obstetric danger signs and having a BPCR plan are the first steps in the appropriate and timely referral to essential obstetric care and part of Kenya’s national guidelines for quality obstetric and perinatal care, is raising awareness of danger signs during pregnancy and childbirth as well as women developing an individualized BPCR plan [20]. The guideline calls for the need to strengthen focused antenatal care and empower women, men, families, and communities through health education at antenatal clinics to be able to recognize pregnancy-related risks and to take responsibility for developing and implementing appropriate responses.

Pastoralism is a global phenomenon. It is rearing of large number of domestic animals and moving them from one place to the other in search of grazing lands. In Africa, where 66% of the land is used for pastoral production, and is recognized as part of the continent's cultural heritage [21]. Pastoralism is a culture, and a way of life intrinsically and deeply linked to the identity of the individuals and communities that practice it. Given their traditional nomadic lifestyle and climate change, the fact that pastoralists can become internally displaced and vulnerable people with special health needs are often overlooked in many regions and governments of the world including in Kenya [22].

Pastoralist women face immense challenges in developing and implementing BPCR plan due to the inherently migratory and unsettled nature of their communities [18, 23, 24]. These populations navigate harsh and unpredictable environments, contending with persistent water shortages, dwindling pastures for their livestock, rampant animal and human diseases, recurrent severe droughts, famine, floods, desertification due to climate change, insecurity, and displacements caused by armed banditry and cattle rustling [24, 25].

Migration is well-known to be a primary coping strategy for the survival of pastoralists [25]. The migratory nature of pastoralists however has made it difficult for governments and other agencies to provide education and health services to these communities and nomadism has been termed unproductive, wasteful, and primitive [26, 27]. Research on pastoralists’ health remains scarce. The limited studies available highlight severe health challenges, including malnutrition, inadequate housing, and lack of clean drinking water [21]. Pastoralist communities face disproportionately high rates of infectious diseases such as malaria, brucellosis, anthrax and typhoid [21]. Education is hindered by high dropout rates, while antenatal care follow-ups suffer due to frequent migrations [13].

The lack of knowledge about ODs and absence of BPCR plan are critical barriers to reducing maternal mortality and morbidity in Sub-Sahara Africa and Kenya, particularly among pastoralist communities like Pokot [3, 7, 28]. While knowledge of these danger signs is essential for preventing birth complications and improving maternal and child health, little is known about their impact on BPCR among Pokot pastoralists in Kenya. This study, therefore, aims to bridge this knowledge gap by assessing the level of knowledge of ODs and its effects on BPCR among Pokot nomadic pastoralist pregnant women across seven wards in Tiaty Sub-county, Baringo Count, Kenya.

## Methodology

This was cross-sectional, facility-based research and was conducted among 275 mobile Pokot nomadic pastoralist pregnant women who were on their second and third trimesters and attending various government, faith-based, and mobile antenatal clinics in Tiaty Sub-County, Baringo County of Kenya. Tiaty Sub-County is divided into seven geopolitical regions or wards and covers an area of 4516.8 square kilometres. These wards are Tirioko, Koloa, Ribkwo, Silale, Loyamotok, Tangulbei/Korossi, and Churo/Amaya wards. The Sub-County is mainly arid and semi-arid and supports little or no agricultural activity. In terms of communication, it is largely inaccessible, and the infrastructure is inadequate. There are few motorable tracks, and the landscape is dissected by numerous seasonal rivers, hills, plains, and mountains [29, 30].

It is predominantly and almost purely inhabited by the Pokot pastoralist community and the major economic activity is nomadic pastoralism [31] (Loina et al., 2017). Pokot culture is deeply rooted in pastoralism and their attitude and perception of cattle can only be compared to that of gold or money in modern society [29]. The cow, also known as Tany or Chemang‟ any in Pokot is the greatest valued animal and asset on earth.

According to the 2009 Kenya Population and Housing Census [32], the East Pokot district had a population of 133,189 people. 69,889 of the population were men while 63,300 were women. A total of 25,619 of the female population were at reproductive ages of 15–49 years. Pastoralists in this area are in constant transhumance and pregnant women health has particularly become vulnerable to constant locational changes like increased distances burden to health facilities, mandatory re-construction of temporary makeshift houses, and other responsibilities which are culturally deemed for women. They are also exposed to desert heatwaves armed banditry and insecurity. Pregnant pastoralist women walk for hours or days to access reproductive health services. 

### Sample size determination and sampling

The sample size was determined to be 305 by use of the sample size standard formulae as used by Fisher's formula 2003 [33]. However, a 90.1% (*n* = 275) response rate was achieved. A multistage sampling technique was used. Firstly, a purposive sampling of six wards: Kolowa, Tirioko, Ripko, Silale, Loyamorok, and Tangulbei wards was done. Churo ward was left out since the community here was more settled and practiced farming and less pastoralism. Through stratified sampling, the community in each ward was grouped into locations, sub-locations, and villages based on where the pastoralists lived and the number of questionnaires to be administered per ward was calculated based on ward population. Lastly, in the wards, hospitals offering antenatal clinics were identified. In the clinics, a convenience sampling method was used to select pregnant women who were above 18 years old and on their second and third trimesters that the data was collected.

### Data collection tool and data collection process

The researcher adopted standardized BPCR questions from the Johns Hopkins Program for International Education in Gynecology and Obstetrics tool [5, 6] to develop the questionnaire and an observation checklist for the danger signs. Data was collected through a semi-structured pre-tested researcher-administered questionnaire between August and October 2017. The questionnaire was translated into the Pokot local language for simplicity and easier understanding by the local community.

### Data management and analysis

The collected raw data was manually checked daily for completeness after collection. It was then categorized, coded, and entered in Statistical Package for the Social Science (SPSS) version 21 for analysis. Chi-square (X^2^) was used to test the association between categorical variables. *P*-value of < 0.05 was considered significant.

### Measurements

The level of knowledge of key danger signs during pregnancy and delivery was assessed. The danger signs of interest during pregnancy were vaginal bleeding, severe headache, blurred vision, convulsions, swollen hands legs, or face, high fever, loss of consciousness, difficulty breathing, severe body weakness, severe abdominal pain/false labour, and reduced fetal movement. During delivery, the danger signs of interest were excessive bleeding, severe headache, convulsions, fever, loss of consciousness, long hours of labour, and retained placenta.

To measure this, a woman was asked to randomly name any danger signs she knows at the 2 levels. Any woman who easily named between 0–2 danger signs was considered to have low knowledge, while those who named 3–5 danger signs were considered to have moderate knowledge. Finally, any woman who named the above 5 danger signs in each category, was considered to have high knowledge of danger signs.

BPCR is a strategy to promote the timely use of skilled maternal and neonatal care, especially during childbirth, based on the theory that preparing for childbirth and being ready for complications by knowing the danger signs of complications reduces delays in obtaining this care.

In measuring BPCR, a total of 5 dependent variables such as identification of skilled birth attendants, identification of reliable means of transport during emergence, saving money for emergency use, saving of foodstuff, and identification of blood donors were used as variables to measure BPCR. Anybody who responded "YES" to any of the above variables scored 1/5 marks and the 0 mark when responding was "NO".

Furthermore, a BPCR score was obtained using the formula below and then changed to percentages.

$$Transformed\;BPCR=\frac{Score\;of\;BPCR\;factors\;of\;respondents}{total\;available\;factors\;score\;(5)}\ast\;100$$

Furthermore, BPCR was categorized into Poor, Moderate, and High knowledge by calculating percentages of all the variable scores and converting them to percentiles (Poor if score ≤ 25^th^ percentile, Moderate if 25^th^ percentile < score ≤ 75^th^ percentile, and Good if score ˃ 75^th^ percentile).

## Results

### Socio-demographic and socio-economic characteristics of the respondents

A total of 275 women who were in their second and third trimesters were interviewed for this study making a response rate of 90.1%. Respondents'ages ranged from 18–44 years with the majority 81.5% being of ages between 18–34 years. Two hundred and sixty-four (96%) were from the pastoralist Pokot ethnic community. Regarding religion, the highest number of the respondents 62.9% (*n* = 173) practiced African Traditional Religion while 36.4% (97) and 0.7% (2) respondents practiced Christianity and Islam respectively. Many of the participants, 95.6% (263) were married and 4.6% (12) were unmarried. Two hundred and twenty-six (82.2%) of the respondents never went to school and thus did not have any formal education compared to 16.8% who at least had some form of formal education. Among those who had formal education, only 2.2% (*n* = 6) completed both secondary and post-secondary education. Furthermore, the research sought to find out the socioeconomic characteristics of the respondents. The findings showed that the highest number of women 91.3%(*n* = 251) did not have any other work to do other than housework. However, they had livestock in their families which acted as their source of income. Families who reported keeping livestock were 94.5% (*n* = 260).

### Obstetric characteristics of the respondents

A total of 154 (56. %) of the respondents were in the second trimester while 121 (44.0%) were in their third trimester. Eighty-nine per cent (239) of the respondents knew only their month of delivery, 3.3% knew their exact date and month of delivery, and 9.8% knew neither the month nor exact date of delivery. Forty (14.5%) were prime gravidae while 235 (85.5%) were multipara. 81.7% (192) of multi-paras had attended ANC in their previous pregnancy while 18.3% (43) did not. Among the respondents'ANC attendance, the majority 56% (145) were attending ANC for the first time. Those who had met the World Health Organization (WHO)-recommended ANCs (at least four ANC visits) were 10.2% (28). A larger number of the respondents 82.3% (195) delivered their past babies at home with traditional birth attendance compared to 17.7% (40) who delivered in a health facility with skilled birth attendants. One hundred and forty-nine (62.9%) reported that they did not experience any form of complications during delivery but 37.1% (88) reported to have experienced some form of complications in their previous delivery.

Whether the respondents were planning to deliver in a health facility with a skilled birth attendant, this research found that a large number of respondents, 40.7% (112) had not yet made up their minds on where they would deliver their current pregnancy, 33.8% (93) said they will deliver at home while 25.5% (70) were planning to deliver at a health facility with a skilled birth attendant. Table 1 below shows the obstetric characteristics of the respondents.

**Table 1:** Obstetric characteristics of the respondents

Variable	Frequency (*n* = 275)	Percentages (%)
Gestation period		
*Second trimester*	154	56
*Third trimester*	121	44
Expected date of delivery		
*Know the date of delivery*	9	3.3
*Knows only month of delivery*	239	86.9
*Don't know the date or month*	27	9.8
Was it the participant's first pregnancy		
*Yes*	140	14.5
*No*	235	85.5
ANC attendance in a previous pregnancy		
*Yes*	192	81.7
*No*	42	18.3
Number of present ANC attendance		
*First*	145	52.7
*Second*	56	20.4
*Third*	46	16.7
*Fourth*	25	9.1
*More than 4 times*	3	1.1
Previous place of delivery		
*In a health facility with a skilled birth attendant*	40	17.7
*At home with a traditional birth attendant*	195	82.3
Complications in the previous delivery		
*Yes*	88	37.1
*No*	149	62.9
Plan on where to deliver their current pregnancy		
*In a health facility with a skilled birth attendant*	70	25.5
*At home with a traditional birth attendant*	93	33.8
*Don’t know*	112	40.7

### Knowledge of danger signs during pregnancy and childbirth

**Fig. 1:**
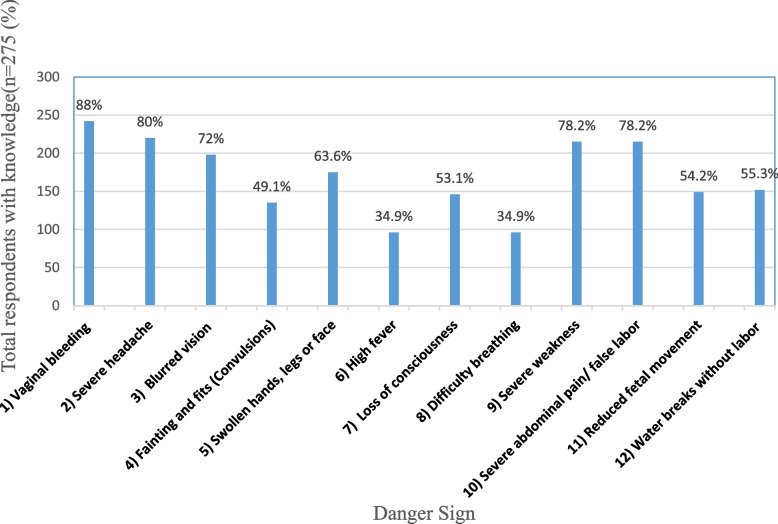
Knowledge of danger signs during pregnancy (*n* = 275)

The results (Fig. 1) below showed that, more than three-quarters of pastoralist women had the highest knowledge on per vaginal bleeding (88%), severe headache (80%), severe body weakness (78.2%), and severe abdominal pain (78.2%) as danger signs during pregnancy. Furthermore, more than half of the respondents indicated that blurred vision (72%), swollen body (63.6%), false labour (55.3%), reduced fetal movement (54.2%) and loss of consciousness (53.1%) were danger signs during pregnancy. However, less than half of the respondents knew fainting, high fever, and difficulty breathing as danger signs during pregnancy.

Knowledge of danger signs during labour (Table 2), a larger majority of the respondents had a high knowledge of danger signs during labour. Severe bleeding after delivery was widely held by the majority 92.4% (254) as a danger sign during labour. The other danger signs mentioned by the respondents were: prolonged labour 85.8% (236), retained placenta 75.6% (208), convulsions 63.3% (175), severe headache 49.8% (137), and fever 30% (83).

**Table 2:** Knowledge of danger signs during labour/delivery (*n* = 275)

Variable	Frequency *n* = (275)	Percentages %
Excessive bleeding	254	92.5
Severe headache	136	49.5
Fits (convulsions)	175	63.6
High body hotness	83	30.2
Loss of consciousness	199	72.4
Long hours of labour (more than 12 h)	236	85.8
Placenta not delivered after baby delivery	208	75.6

Knowledge of danger signs at the two levels was summarized into low, moderate, and high knowledge. The findings showed that the majority of the respondents had high knowledge of obstetric danger signs at the two levels as shown in Table 3 below.

**Table 3:** Level of knowledge of danger signs during pregnancy & childbirth

Variable	Low (%)	Moderate (%)	High (%)
Knowledge of danger signs during pregnancy	36(13.1%)	19(6.9%)	220(80.0%)
Knowledge of danger signs during labour	44(16.0%)	41(14.9%)	190(69.1%)

### Birth preparedness and complications readiness

**Fig. 2:**
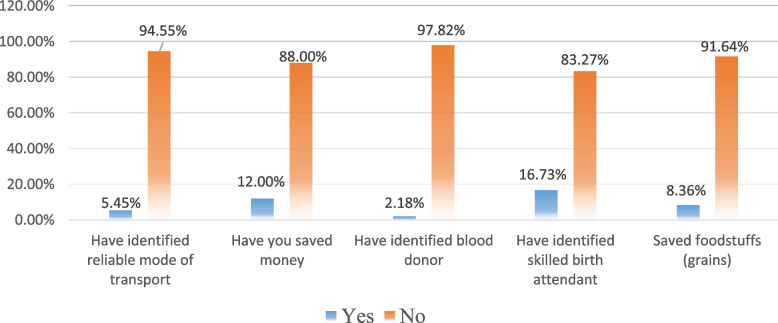
Birth Preparedness and Complications Readiness of the respondents(*n* = 275)

The study findings (Fig. 2) showed that 5.45% of the respondents had identified reliable modes of transport, 12% had saved money, 2.18% had identified blood donors, 16.73% had identified skilled birth attendants and 8.36% had saved foodstuffs (grains).

**Fig. 3:**
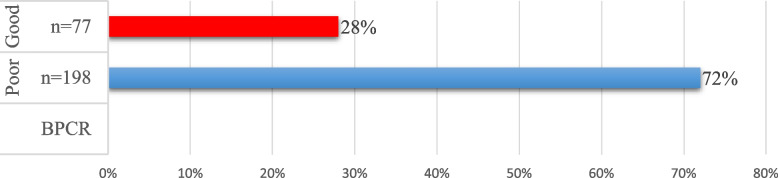
Levels of BPCR of the respondents

When percentiles (Fig. 3 below) were used to identify the level of BPCR, it was found that a total of 198 (72%) of the respondents were poorly prepared for birth and complications while 77 (28%) had prepared well for birth and complications. There were no respondents in the moderate category.

### Association between knowledge of obstetric danger signs and birth preparedness and complications readiness

The findings showed that knowledge of danger signs at all levels (pregnancy (X^2^ = 0.80, *P* = 0.67) and at childbirth (X^2^ = 0.46, *P* = 0.80) had no significant association with BPCR as shown by Table 4 below. The majority of the respondents had a high knowledge of danger signs during pregnancy and childbirth. However, having high knowledge did not make the respondents prepare for birth and complications.

**Table 4:** Association between knowledge of obstetric danger signs and birth preparedness and complication readiness

**Variables**	**BPCR**		${{\varvec{X}}}^{2}$	***P* value**
	**Poor (%)**	**Good (%)**		
**Danger signs during pregnancy**				
*0–2 (Low)*	26(13.1)	10(13.0)	0.80	0.67
*3–5 (Moderate)*	12(6.1)	7(9.1)		
*5 and above*	160(80.8)	60(77.9)		
**Danger signs during labour**				
*0–2 (Low)*	31(15.7)	13(16.9)	0.46	0.80
*3–5 (Moderate)*	28(14.1)	13(16.9)		
*5 and above*	139(70.2)	51(66.2)		

## Discussion

Knowledge of obstetric danger signs during pregnancy and labour is the first essential step for appropriate safe motherhood, especially in remote Sub-Sahara Africa where the shortage of medical personnel is high [1, 3, 7]. The objective of this study was to assess the knowledge of danger signs during pregnancy and childbirth and determine its effect on BPCR.

Complications during pregnancy and childbirth arise due to the failure of the individual women to identify these impending dangers and the failure to take necessary steps to avert these problems [34, 35]. Many lives have been lost due to a lack of knowledge and failure to recognize threats posed by these danger signs at different levels of pregnancy. This has led to a time increase in the three public health delays in seeking health care hence Kenya’s high MMR.

This study showed mixed findings among the pastoralist Pokot Nomadic community. The results showed that the respondents had a high knowledge of obstetric danger signs at the two levels of pregnancy and childbirth. Vaginal bleeding, severe headache, severe body weakness, and severe abdominal pain were dominantly known by the women as danger signs during pregnancy. These findings were consistent with findings found by Desta Hailu &Hailemarium and those of Mesay Hailu and Abebe Gebremariam in research done in the Tsegedie and Wondo districts of Ethiopia.

High knowledge of danger signs during delivery was also witnessed among the respondents with excessive bleeding and prolonged labour being widely known.

This high knowledge of danger signs might be attributed to high ANC attendance in the area. The respondents who reported having attended clinic in their previous pregnancy were 81.7% (226) and those who reported that the present ANC was not their first time attending ANC was 85.5% (235). This research confirms that Pokot pastoralist women attend ANC. Kenya's government through the Ministry of Health and Beyond Zero Campaigns together with non-governmental organizations which were working in the area at the time of the study like Kenya Red Cross, World Vision, and East Pokot Medical Project conducted a series of mobile clinics in the area targeting pastoralists.

This therefore could be the major reason as to why the respondents had good knowledge of danger signs. These findings compare well with findings from the Kenya Demographic Health Survey of 2014 where it was reported that 92% of Kenya women receive antenatal care at least once in their pregnancies [36, 37] and that of research done at Kenyatta National Hospital Kenya [10], where it was found that women attending Kenyatta National hospital had high knowledge of danger signs. The research also supports research done in Wareng District Kenya, where pregnant women had high ANC attendance and high knowledge of danger signs [38]. Attendance of antenatal clinics gives women the chance to receive health education which comprises knowledge of danger signs and birth preparedness [28].

The major findings that this study highlighted, are on the association between knowledge of obstetric danger signs and its effects on BPCR among the Nomadic Pokot pastoralist community. The findings of the study showed a complete lack of association. In a normal context, high knowledge would have been associated with better preparedness for better maternal and child outcomes. However, respondents in this study had high knowledge of danger signs at all levels (during pregnancy and delivery), but BPCR was extremely low (28%). Less than a quarter of the respondents had put in place BPCR measures that were under study (identification of reliable mode of transport, saving emergency funds, identifying blood donors, saving foodstuffs, and identifying reliable skilled birth attendants and hospitals). This is a red flag in the fight against the eradication of maternal and infant mortality among pastoralist communities who are in constant movement for their survival and that of their animals in arid and harsh environments in Kenya.

This study recommends well-organized and coordinated mobile clinics for pastoralists by all stakeholders and simplified health education reproductive health programs on BPCR, Danger signs, hospital delivery, immunization, male involvement, family planning, early marriage, and youth reproductive health should be offered.

Government should not only emphasize the cost of delivery but also health information access and availability of services, especially in pastoral communities. As part of reducing maternal and infant mortality, Knowledge of OD and BPCR should strongly be emphasized. BPCR ensures that deaths resulting due to poor planning for delivery and emergencies are avoided. Delays in seeking health care are also prevented. Finally, special maternity homes (waiting homes) should be built in designated pastoral hospitals in the area. These homes could serve as waiting areas for the pastoralists'women when they are in their last months of pregnancy.

## Limitations

This study exclusively focused on mobile Pokot nomadic pastoralist pregnant women in Tiaty Sub-County, Baringo County-Kenya, which may limit the generalization of the findings to other populations, including settled communities or different pastoralist groups with varying cultural and healthcare access factors. The study also relied on self-reported data, which may introduce recall bias or social desirability bias, as participants may have provided responses they believed to be favourable. The cross-sectional design only provides a snapshot of knowledge and preparedness at a single point in time, making it difficult to assess changes over time or establish causal relationships.

## Conclusion

Despite high knowledge of obstetric danger signs among Pokot pastoralist women, BPCR remain critically low. This gap presents both a challenge and an opportunity. Maternal mortality can significantly be reduced if healthcare services are made accessible, available and affordable. The government and stakeholders must implement bold, targeted interventions, including robust mobile clinics, community-based birth preparedness programs, emergency transport networks, maternity waiting homes, improved referral systems, culturally sensitive health education, and strategic policy and infrastructure investments. Prioritizing these community-centered solutions will transform maternal health outcomes and save lives among Kenya’s pastoralist populations.

## Data Availability

The datasets supporting the conclusions of this article are not publicly available but are available from the corresponding author on reasonable request.

## Abbreviations

ANC: Antenatal Care
BPCR: Birth Preparedness and Complications Readiness
IOM: International Organization for Migration
IREC: Institutional Research and Ethics Committee
JHPIEGO: Johns Hopkins Program for International Education in Gynecology and Obstetrics
KNBS: Kenya National Bureau of Statistics
MM: Maternal Mortality
MMR: Maternal Mortality Ratio
MoH: Ministry of Health
ODS: Obstetric Danger Signs
UN: United Nations
UNICEF: United Nations International Children’s Emergency Fund
UNFPA: United Nations Population Fund
WHO: World Health Organization
